# The relationship between treatment motivation, coping, and psychosocial functioning in a schizophrenia patient’s sample

**DOI:** 10.1192/j.eurpsy.2022.1962

**Published:** 2022-09-01

**Authors:** N. Semenova, B. Kazakovtsev, M. Kachaeva, L. Burygina

**Affiliations:** 1 Moscow Research Institute of Psychiatry, Clinical Psychology Counseling, Moscow, Russian Federation; 2 V. Serbsky National Medical Research Centre for Psychiatry and Narcology, Department Of Epidemiological And Organizational Problems Of Psychiatry, Moscow, Russian Federation; 3 V. Serbsky National Medical Research Centre for Psychiatry and Narcology, Forensic Psychiatry, Moscow, Russian Federation; 4 Psychiatric hospital No. 4 named after P.B. Gannushkin, Administration, Moscow, Russian Federation

**Keywords:** Psychosocial functioning, coping, schizophrénia, Treatment motivation

## Abstract

**Introduction:**

Schizophrenia is characterized by impairments in motivation and coping and decrements in psychosocial functioning in major life areas.

**Objectives:**

This study attempted to examine the links between treatment motivation, coping, and psychosocial functioning for persons with schizophrenia. Design: Cross-sectional survey.

**Methods:**

One hundred thirty-eight participants were recruited at random from outpatient psychosocial rehabilitation programs in Moscow-based psychiatric hospitals. The measures of motivation were administered by testers blind to scores on other study variables; measures of coping (COPE, CERQ) and psychosocial functioning (PSP, EQ5D5L, SF36, Q-Les-Q-18) were administered. Data were analyzed using latent construct modeling to test for mediator and moderator effects.

**Results:**

There were strong bivariate relationships between coping, motivation, and psychosocial functioning. The results demonstrated that coping strongly mediated the relationship between motivation and psychosocial functioning. This mediation was evidenced by: 1) the direct path from motivation to a functional outcome no longer being statistically significant after introducing coping into the model; 2) the statistical significance of the indirect path from motivation through coping to functional outcome. There was no support for the moderation hypotheses.

**Conclusions:**

Motivation influences psychosocial functioning through its relationship with coping, and coping is a critical mechanism for explaining the relationship between motivation and psychosocial functioning. These results will be compared with work on motivation, neurocognition, and psychosocial functioning in schizophrenia (Nakagami et al. 2008), as well as with gender issues. Professionals working with schizophrenia patients should consider such variables as coping when designing and implementing gender-sensitive intervention programs.

**Disclosure:**

No significant relationships.

